# Airway epithelial‐derived exosomes induce acute asthma exacerbation after respiratory syncytial virus infection

**DOI:** 10.1002/mco2.621

**Published:** 2024-06-27

**Authors:** Ye Yao, Yu Yang, Ming Ji, Qingwu Qin, Kun Xu, Zhenkun Xia, Huijun Liu, Lin Yuan, Yunchang Yuan, Ling Qin, Xizi Du, Leyuan Wang, Kai Zhou, Xinyu Wu, Weijie Wang, Bei Qing, Yang Xiang, Xiangping Qu, Ming Yang, Xiaoqun Qin, Chi Liu

**Affiliations:** ^1^ Department of Respiratory Medicine National Clinical Research Center for Respiratory Diseases Xiangya Hospital Central South University Changsha China; ^2^ Department of Physiology School of Basic Medicine Science Central South University Changsha Hunan China; ^3^ Department of Pulmonary and Critical Care Medicine the Second Xiangya Hospital Central South University Changsha China; ^4^ Department of preventive medicine, School of Medicine Hunan Normal University Changsha China; ^5^ Department of Thoracic Surgery the Second Xiangya Hospital Central South University Changsha Hunan China; ^6^ Basic and Clinical Research Laboratory of Major Respiratory Diseases Central South University Changsha Hunan China; ^7^ Centre for Asthma and Respiratory Disease School of Biomedical Sciences and Pharmacy Faculty of Health and Medicine University of Newcastle and Hunter Medical Research Institute Callaghan New South Wales Australia

**Keywords:** acute asthma exacerbation, airway epithelial cells, exosomes, respiratory syncytial virus, Th2 inflammation

## Abstract

Acute asthma exacerbation refers to the progressive deterioration of asthma symptoms that is always triggered by virus infection represented by respiratory syncytial virus (RSV). After RSV infection, exaggerated Th2‐mediated pulmonary inflammation is the critical pathological response of asthmatic patients with acute exacerbation. Significantly, airway epithelial cells, being the primary targets of RSV infection, play a crucial role in controlling the pulmonary inflammatory response by releasing airway epithelial cell‐derived exosomes (AEC‐Exos), which potentially influence the development of asthma. However, the specific role of AEC‐Exos in acute asthma exacerbation after RSV infection remains obscure. The purpose of this study was to determine the distinct function of AEC‐Exos in exacerbating acute asthma following RSV infection. Blockade of exosomes by GW reduce the enhanced pulmonary inflammation significantly. Specifically, the enhanced Th2 inflammation was induced by AEC‐Exos thorough transportation of hsa‐miR‐155‐5p–Sirtuin 1 (SIRT1) pathway during acute asthma exacerbation. Targeted inhibition of hsa‐miR‐155‐5p blocks the exaggerated Th2 inflammation effectively in mice with acute asthma exacerbation. In summary, our study showed that during acute asthma exacerbation after RSV infection, AEC‐Exos promote the enhanced Th2 inflammation through transportation of increased hsa‐miR‐155‐5p, which was mediated partly through SIRT1‐mediated pathway. hsa‐miR‐155‐5p is a potential biomarker for early prediction of acute asthma exacerbation.

## Graphical abstract

Acute asthma exacerbation is sudden or aggravated asthma symptoms in asthma patients, which is induced by outside stimulants. During acute asthma exacerbation after RSV infection, AEC‐Exos promote the enhanced Th2 inflammation by transporting increased hsa‐miR‐155‐5p, which was mediated partly through SIRT1‐mediated pathway. hsa‐miR‐155‐5p is a potential biomarker for early prediction of acute asthma exacerbation.

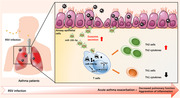

## 1 INTRODUCTION

Acute asthma exacerbation are sudden or aggravated asthma symptoms (shortness of breath, cough, chest tightness, etc.) in asthma patients, which is always induced by outside stimulants (i.e. allergens, respiratory viruses).^1^ Epidemiological data demonstrate that the prevalence and mortality of acute asthma exacerbation is increasing obviously in recent years, which has become the leading cause of harm to the health and financial burden of asthma patients.^2^, ^3^, ^4^, ^5^ However, the traditional low‐ and medium‐dose of inhaled corticosteroids (ICS) has no dramatic therapeutic effect on acute asthma exacerbation, which usually requires higher dose ICS and additional control drugs to alleviate symptoms. Consequently, systemic ICS are administrated for at least 3 days for the regular treatment of asthmatic patients with acute exacerbation. Nevertheless, there is still no definite biomarker or effective therapy target for the detection or treatment of acute asthma exacerbation.

Respiratory syncytial virus (RSV) is a common trigger of acute asthma exacerbation. A series of typical pathological characteristics (including enhanced airway inflammation, increased airway hypersensitiveness and serious airway obstruction) were induced in asthma patients with acute exacerbation after RSV infection.^6^ Different from healthy subjects, asthma patients are highly susceptible to RSV and the clearance of RSV is reduced significantly.^7^ In particular, a series of researches have demonstrated that T cells are hyperactivated during acute asthma exacerbation after RSV infection.^8^ Specifically, the enhanced Th2‐type inflammation has been proved to directly induce acute asthma exacerbation after RSV infection, highlighting the pivotal role of enhanced Th2 inflammation in exacerbating acute asthma.^9^, ^10^ As the initial target cells for RSV infection, airway epithelial cells (AECs) play a critical role in the immune process after RSV infection in asthma patients.^11^ Increasing evidence indicates that AECs play a crucial role in regulating the pulmonary immune response by releasing exosomes, which in turn promote the initiation and progression of asthma.^12^, ^13^

Exosomes are a kind of extracellular vesicles with a diameter of 30−150 nm, that is secreted by various cells including epithelial cells.^14^ By transmitting effective biological information between distinct cell types, exosomes were involved in intercellular communication and many pathophysiological processes, including immune response, neurodegeneration, virus infection, and so on.^15^, ^16^ It is noteworthy that, AEC‐derived exosomes (AEC‐Exos) are extremely sensitive to external stimuli, which are important player in the pathological process of chronic pulmonary inflammatory disease (i.e., asthma and COPD).^17^ Specially, significant differences in the phenotype and function of exosomes were detected in the BALF of asthma patients which may influence the development of asthma patients.^18^, ^19^ However, the specific function of AEC‐Exos in asthma is still obscure.

This study was implemented to further explore the effect of AEC‐Exos on the pulmonary inflammation and subsequent acute exacerbation of asthma. First, the mice model of acute asthma exacerbation was constructed to verify the engagement of exosomes in acute asthma exacerbation. Second, the differential miRNA in the exosomes derived from AECs in mice with acute asthma exacerbation was screened and validated. Third, the impact of the increased hsa‐miR‐155‐5p in AEC‐Exos and the specific molecular mechanism on the activation of T cells was investigated. Finally, hsa‐miR‐155‐5p are effectively blocked to clarify the direct effect of hsa‐miR‐155‐5p on the enhanced Th2 inflammation and acute asthma exacerbation. It was determined that AEC‐Exos played a role in the excessive pulmonary inflammation during acute asthma exacerbations, offering new insights and strategies for the early detection and management of acute asthma exacerbations.

## 2 RESULTS

### 2.1 Involvement of exosomes in the enhanced inflammation of mice with acute asthma exacerbation after RSV infection

**FIGURE 1:**
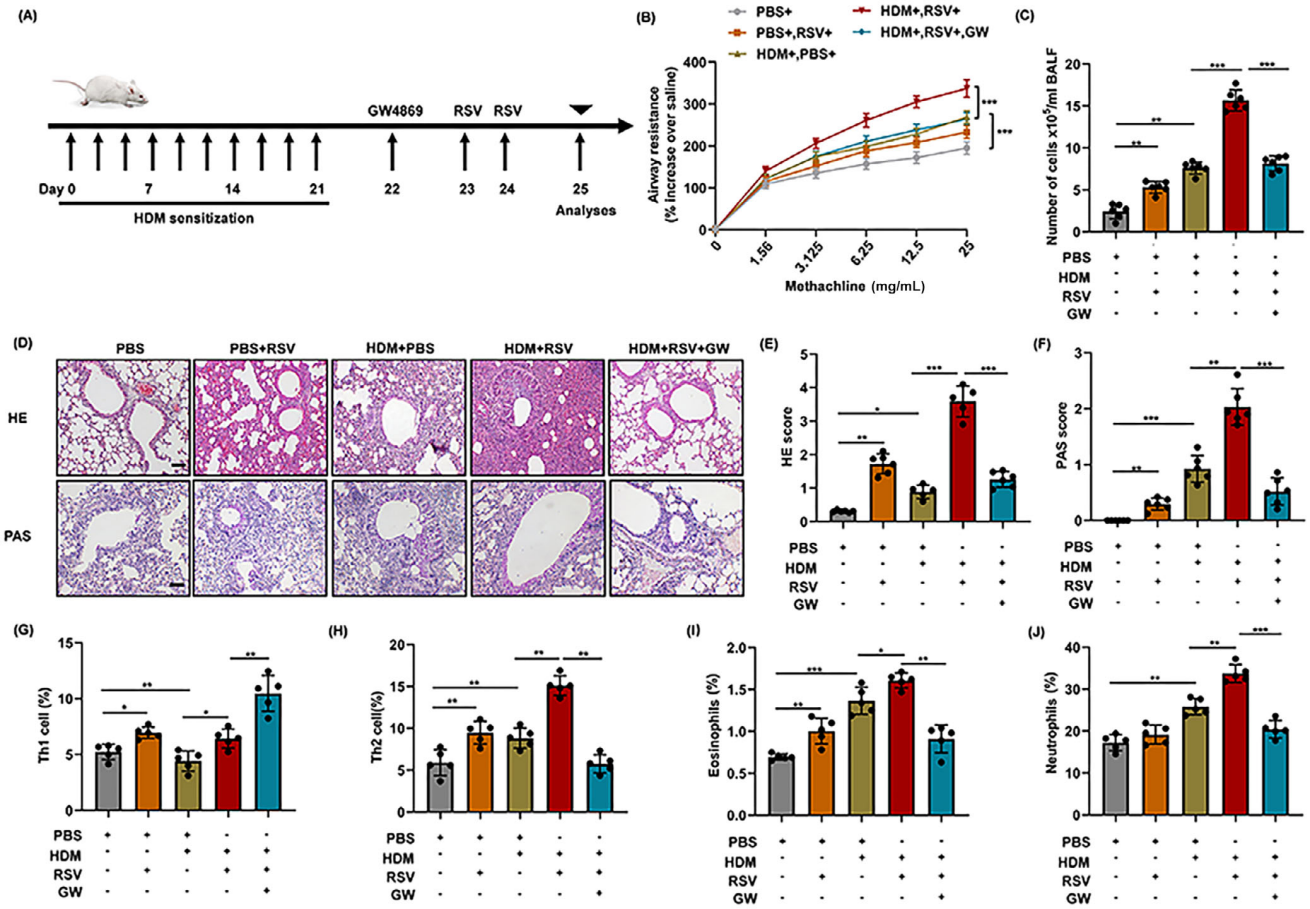
Involvement of exosomes in the enhanced pulmonary inflammation in asthmatic mice with acute exacerbation. (A) Mice were sensitized to HDM/RSV on days 0, 7, 14, 21, 23, or 24. Some mice were treated with GW4869 to specifically delete exosomes in vivo. (B) AHR was represented as airway resistance to increasing doses of methacholine. (C) Inflammatory cell in BALF was counted (*n* = 6). (D) H&E staining and PAS staining of lung tissue (*n* = 6), bars = 50 µm. (E and F) Histopathology score. (G–J) The frequency of Th1 cells, Th2 cells, eosinophils, and neutrophils in BALF (*n* = 5). Data show means ± SDs and are pooled from three independent experiments with four to six samples in each group. *p < 0.05; **p < 0.01; ***p < 0.001.

HDM stress combined with RSV infection was used to establish the mice model of acute asthma exacerbation (Figure 1A). HDM stress induced more significant airway hyperresponsiveness (AHR) than control mice. Moreover, more severe AHR is induced after HDM stress combined with RSV infection (Figure 1B). Meanwhile, increased total cells in BALF, aggravated lung inflammation, and enhanced mucus secretion were induced after HDM stress. Of note, the inflammation degree and the secretion of mucus in mice with acute asthma exacerbation were dramatically serious (Figure 1C,D). In line with the aforementioned findings, the inflammation infiltrates in the lungs of acute asthma exacerbation group also augmented significantly compared with asthma group (Figure 1D–F). Specifically, the elevated inflammatory cells are mainly composed of Th2 cells, eosinophils, and neutrophils (Figure 1G–J).

To further verify the possible role of exosomes in the exaggerated inflammation, GW was used to block the secretion of exosomes in mice with acute asthma exacerbation. Consistent with our conjecture, inhibition of exosomes significantly decreased the level of lung inflammation in mice with acute asthma exacerbation (Figure 1C,D). Meanwhile, exaggerated AHR and increased mucus secretion were also inhibited significantly after exosomes blockade in acute asthma exacerbation group, as compared with asthma group (Figure 1B,D). Flow cytometry analysis demonstrated that treatment with GW significantly decreased the percentage of Th2 cells, eosinophils, and neutrophils, while increasing the percentage of Th1 cells (Figure 1G–J). In particular, the reduction in Th2 cells is more significant than the decreases in eosinophils and neutrophils. Scatter charts are displayed in the Supporting Information (Figure S1A). Flow cytometry further revealed that GW treatment significantly reduced the proportion of Th2 cells, eosinophiles, and neutrophiles, while increased the proportion of Th1 cells (Figure 1G–J). In particular, the reduction in Th2 cells was significantly more pronounced compared with the decrease in eosinophils and neutrophils. Scatter charts are presented in Supporting Information (Figure S1A).

Similarly, the mRNA levels of cytokines associated with T‐cell subsets post differentiation showed a significant increase in the expression of IL‐4, IL‐5, and IL‐13 in the lungs of asthmatic mice, accompanied by a decrease in IFN‐γ expression. Moreover, the level of Th2 cytokines (IL‐4 and IL‐5) in the mice with acute asthma exacerbation increased more obviously which was blocked by GW administration (Figure S1C,D). These results preliminarily infer that exosomes are involved in the regulation of pulmonary inflammation in mice with acute asthma exacerbation, especially the activation and differentiation of T cells. Together, these data provide evidence that exosomes play an important role in the regulation of Th2‐dominated pulmonary inflammation in acute asthma exacerbation.

### 2.2 Isolation and identification of exosomes

**FIGURE 2:**
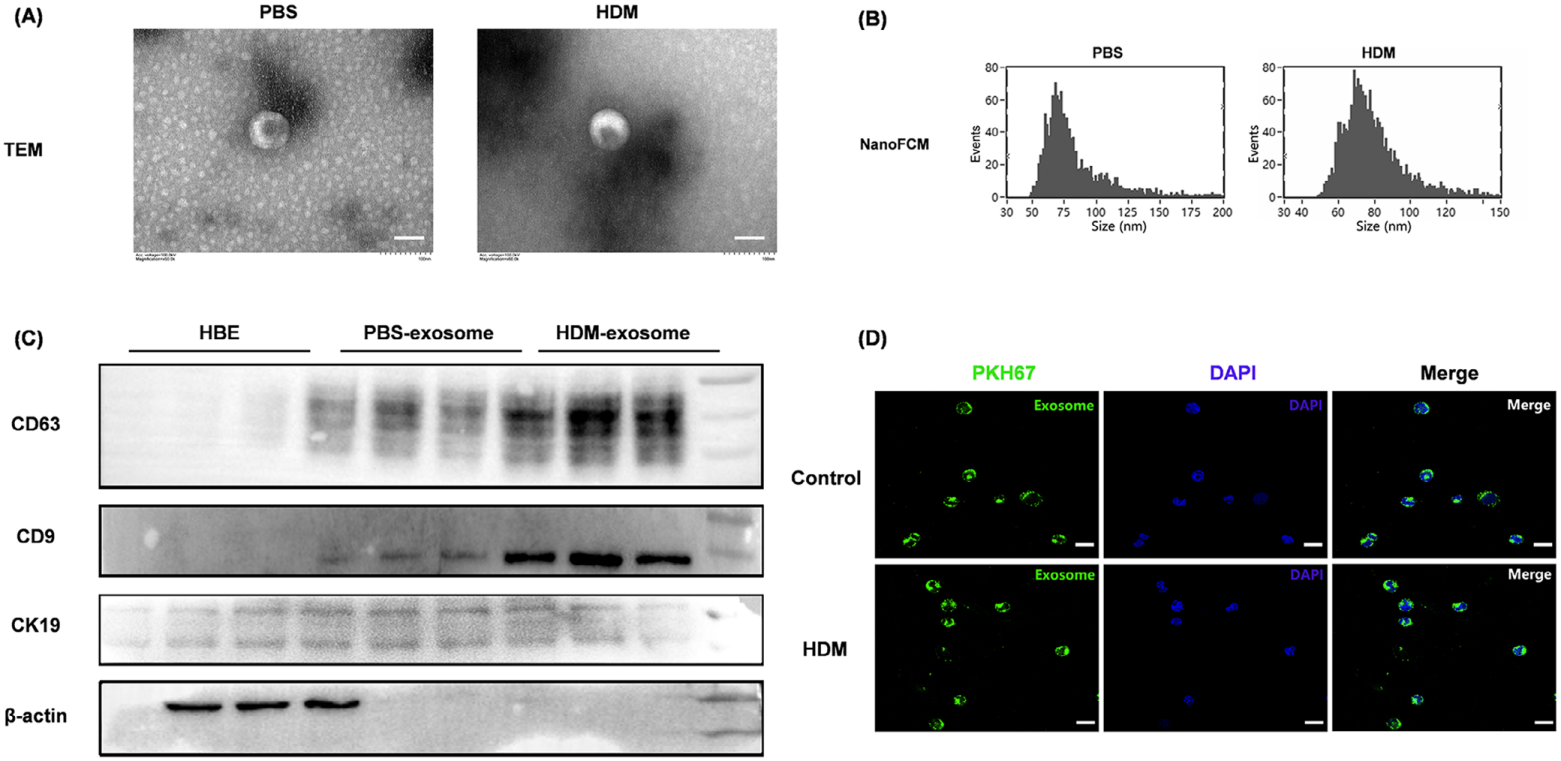
Isolation and identification of exosomes. (A) Characterization of exosomes morphology by TEM, bars = 100 nm. (B) The concentration and diameter of exosomes were measured by FNA. (C) Detection of exosome marker proteins (CD63 and CD9) by Western blot. (D) The uptake of AEC‐Exos by CD4^+^ T cells was detected by confocal microscopy, bar = 10 µm. *p < 0.05; **p < 0.01; ***p < 0.001.

The separated exosomes from the supernatant of AECs were identified by Transmission Electron Microscope (TEM), nanoflow analysis, and western blotting, respectively. Our results demonstrated that the isolated AEC‐Exos showed characteristic vesicle morphology (Figure 2A), with a diameter of 50−110 nm (Figure 2B). At the same time, the localization of exosome marker proteins (CD63 and CD9) was also indicated (Figure 2C). Of note, the expression of CD63 and CD9 from AEC‐Exos increased significantly after HDM stress, suggesting that allergen stress induces the derivation of AEC‐Exos.

Then, to further confirm the interaction between AEC‐Exos and CD4^+^ T cells, PKH67‐labeled AEC‐Exos and CD4^+^ T cells were cocultured. Twenty‐four hours after coculture, AEC‐Exos was absorbed by CD4^+^ T via endocytosis. In addition, no statistical difference was detected in the rate and quantity of exosome uptake by CD4^+^ T cells after HDM stress (Figures 2D and S2).

### 2.3 AEC‐Exos enhance the proliferation and differentiation of CD4^+^ T cells

**FIGURE 3:**
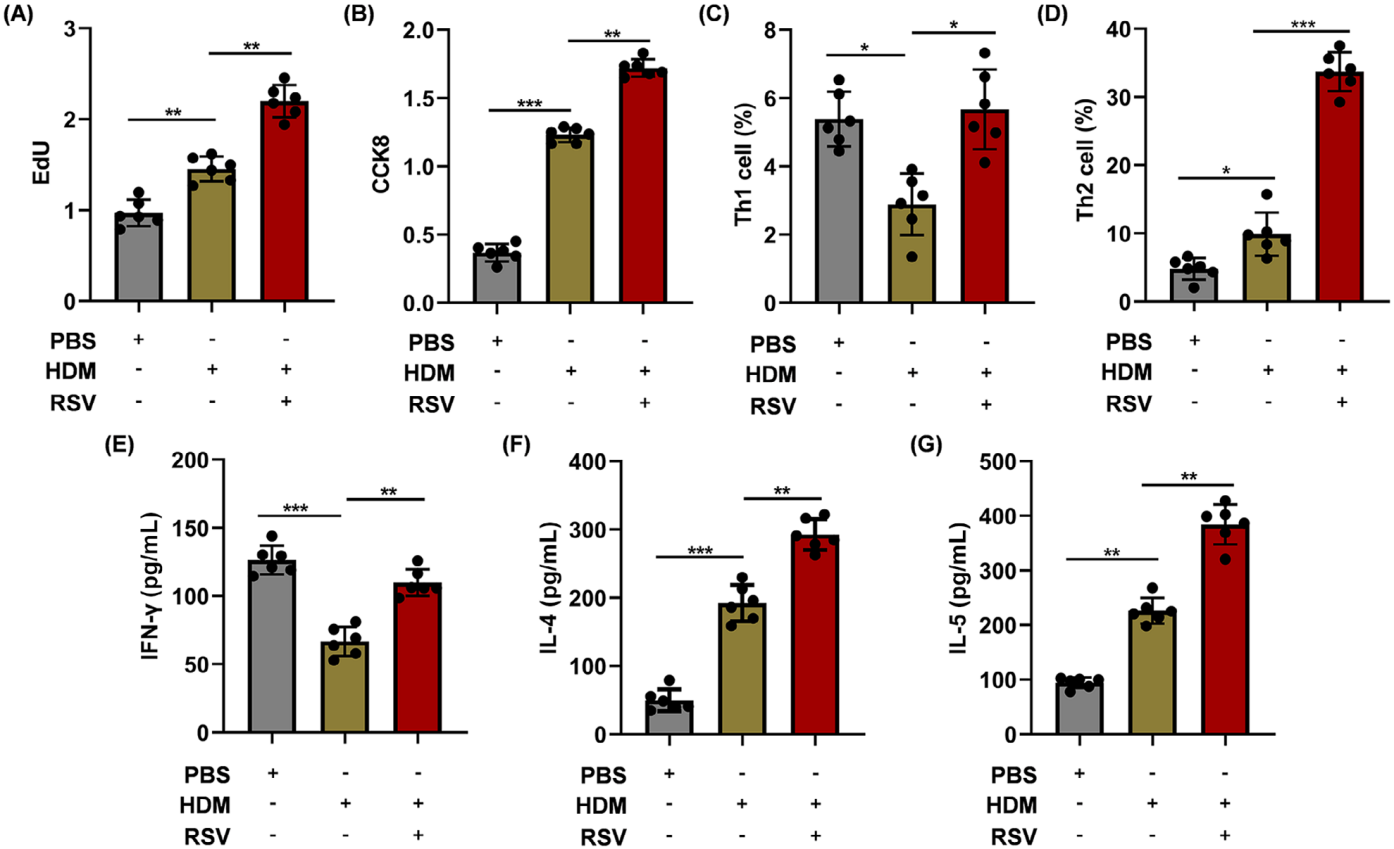
The proliferation and differentiation of CD4^+^ T lymphocytes was regulated by AEC‐Exos. (A and B) The proliferation of CD4^+^ T cells was detected by EdU and CCK‐8, respectively. (C and D) The frequency of Th1 cells and Th2 cells was detected by flow cytometry (*n* = 6). (E–G) The level of IFN‐γ, IL‐4, and IL‐5 in the supernatant were detected by ELISA (*n* = 6). *p < 0.05; **p < 0.01; ***p < 0.001.

In order to assess the capacity of AEC‐Exos to enhance the proliferation and differentiation of CD4^+^ T cells, AEC‐Exos were isolated and incubated with CD4^+^ T cells for a period of 5 days. After coculture, the proliferation of CD4^+^ T cells increased in the HDM group and the proliferation is most prominent in the HDM+RSV group (Figure 3A,B). To further explore the effect of AEC‐Exos on the differentiation of CD4^+^ T cells, the subsets of Th1 and Th2 cells after coculture were detected by flow cytometry. The preprocessing of AEC‐Exos greatly promoted the differentiation of CD4^+^ T cells into Th2 cells and simultaneously suppressed the differentiation of Th1 cells. Moreover, compared with HDM group, the differentiation of Th2 cells increased more obviously in HDM+RSV group, while the proportion of Th1 cells increased slightly (Figures 3C,D and S3A). Furthermore, the mRNA levels in CD4^+^ T cells indicated an upregulation in the expression of Th1 cytokine IFN‐γ, as well as Th2 cytokines IL‐4 and IL‐5, in the group exposed to HDM+RSV when contrasted with the group exposed to HDM. The increase of IL‐4 and IL‐5 is more obvious than that of IFN‐γ (Figure S3B–D). Consistent with the mRNA results, the expression of these cytokines (IFN‐γ, IL‐4, and IL‐5) in the supernatant of CD4^+^ T cells demonstrated a consistent trend (Figure 3E–G). Altogether, these data provide evidence that, after combined stress with HDM and RSV, AEC‐Exos promote the differentiation of CD4^+^ T cells into Th2 cells.

### 2.4 AEC‐Exos induce the enhanced Th2‐type inflammation by transporting hsa‐miR‐155‐5p–Sirtuin 1 axis

**FIGURE 4:**
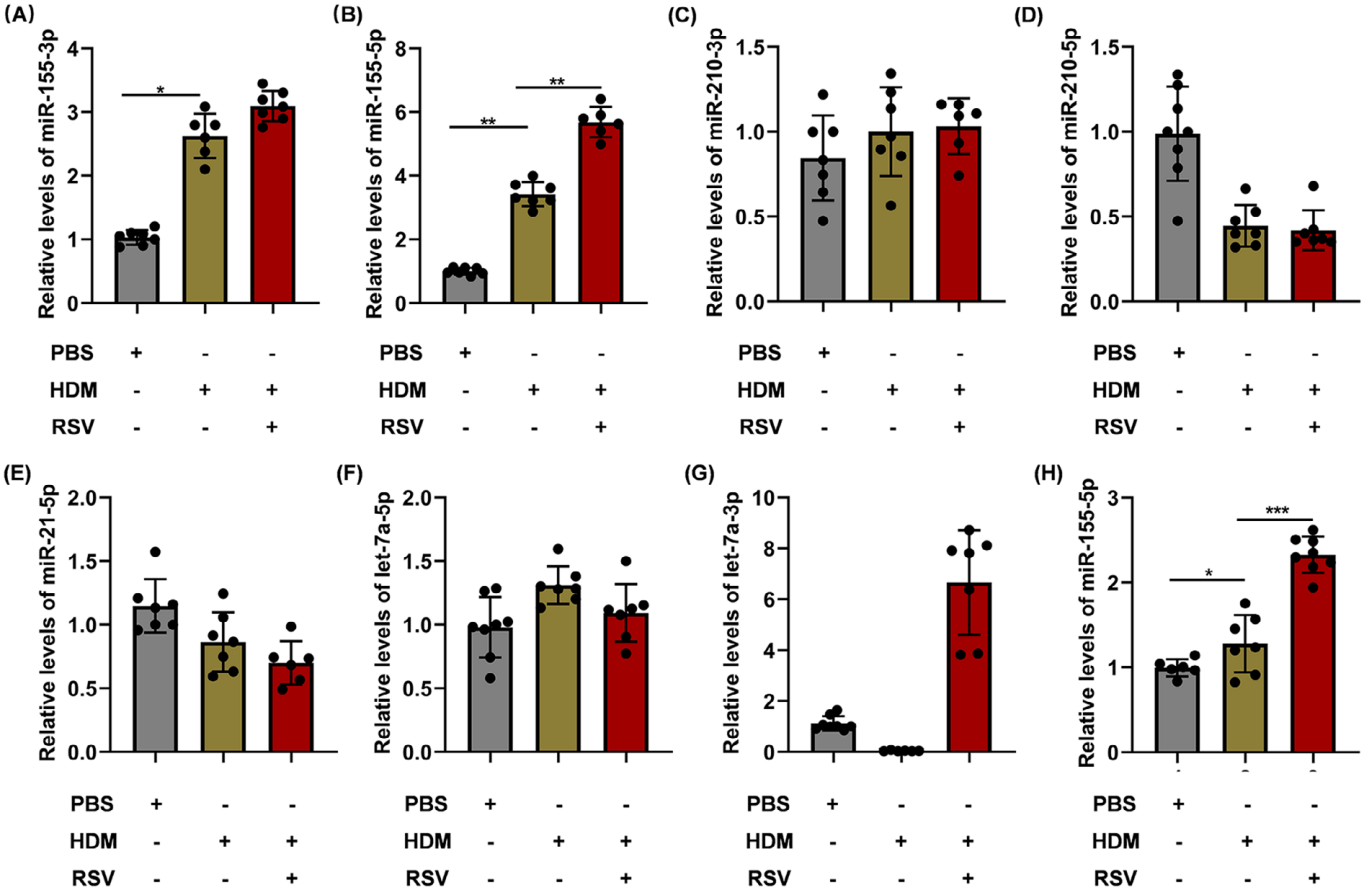
Screening of candidate key miRNAs. (A–G) The mRNA expression of hsa‐miR‐155‐3p, hsa‐miR‐210‐5p, hsa‐miR‐155‐5p, hsa‐miR‐210‐3p, hsa‐let‐7a‐2‐3p, hsa‐let‐7a‐5p, and hsa‐miR‐21‐5p was detected by miRNA RT‐qPCR. (H) The expression of hsa‐miR‐155‐5p in the lungs of mice with acute asthma exacerbation by miRNA RT‐qPCR (*n* = 7). *p < 0.05; **p < 0.01; ***p < 0.001.

To explore the idea that certain specific miRNA in exosomes may be involved in the intercellular signaling to modulate the development of enhanced Th2 inflammation in acute asthma exacerbation, miRNAs was screened and assessed from AEC‐Exos. We discovered nine miRNAs (hsa‐miR‐155‐3p, hsa‐miR‐210‐5p, hsa‐miR‐21‐5p, hsa‐miR‐155‐5p, hsa‐miR‐21‐3p, hsa‐miR‐210‐3p, hsa‐let‐7a‐2‐3p, hsa‐let‐7a‐5p, and hsa‐let‐7a‐3p) that are closely associated with pulmonary inflammation in asthma. Only hsa‐miR‐155‐3p and hsa‐miR‐155‐5p exhibited a significant upward trend in the exosomes of the HDM group and HDM+RSV group, as depicted in Figure 4A–G (hsa‐miR‐21‐3p and hsa‐let‐7a‐2‐3p are excluded due to their extremely low expression).

**FIGURE 5:**
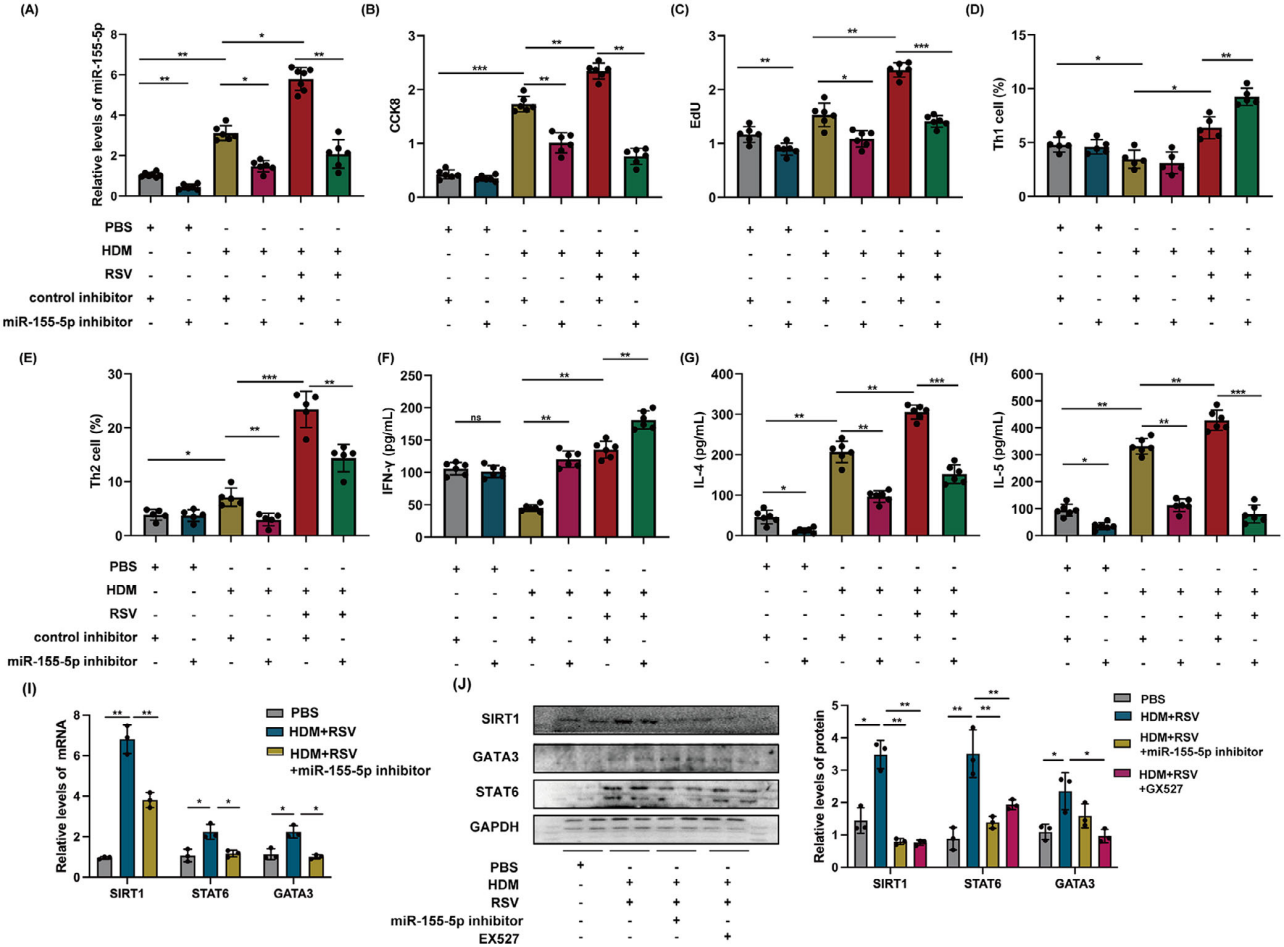
Proliferation and activation of CD4^+^ T cells were induced by hsa‐miR‐155‐5p in AEC‐Exos. (A) The mRNA expression of hsa‐miR‐155‐5p in exosome was identified by miRNA RT‐qPCR (*n* = 6). (B and C) The proliferation of CD4^+^ T cells was detected by EdU and CCK‐8, respectively. (D and E) The frequency of Th1 cells and Th2 cells was detected by flow cytometry (*n* = 6). (F–H) The content of IFN‐γ, IL‐4, and IL‐5 in the supernatant was detected by ELISA (*n* = 6). (I) The mRNA expression of SIRT1, STAT6, and GATA3 in T cells was identified by RT‐qPCR (*n* = 3). (J) The protein level of SIRT1, STAT6, and GATA3 in T cells was identified by Western blot. *p < 0.05; **p < 0.01; ***p < 0.001.

In addition, the expression of hsa‐miR‐155‐5p also increased significantly in the lungs of mice with acute asthma exacerbation (Figure 4H). These findings implied that hsa‐miR‐155‐5p in AEC‐Exos may be a critical candidate that mediates the enhanced Th2 inflammation in acute asthma exacerbation. In this study, hsa‐miR‐155‐5p inhibitor was employed to evaluate the impact of hsa‐miR‐155‐5p, resulting in effective inhibition of hsa‐miR‐155‐5p as shown in Figure 5A. Consistent with our conjecture, the proliferation of CD4^+^ T cells in the HDM+RSV group was suppressed significantly after inhibition of hsa‐miR‐155‐5p (Figure 5B,C). Moreover, the enhanced Th2 cells in the HDM+RSV group was also inhibited and the percentage of Th1 cells increased after inhibiting hsa‐miR‐155‐5p (Figures 5D,E and S4A). Moreover, the upregulated mRNA levels of IL‐4 and IL‐5 in the cocultured CD4^+^ T cells from the HDM+RSV group noticeably decreased, while the expression of IFN‐γ slightly increased following the inhibition of hsa‐miR‐155‐5p (Figure S4B–D). The content of IFN‐γ, IL‐4, and IL‐5 in the supernatant after coculture was consistent with that of mRNA expression (Figure 5F–H). Taken together, these findings indicate that AEC‐Exos promote the enhanced Th2 inflammation by delivering hsa‐miR‐155‐5p to the effector CD4^+^ T cells in acute asthma exacerbation.

Afterward, we investigated the molecular mechanism of hsa‐miR‐155‐5p in regulating Th2 differentiation. As is well known, transcription factors GATA binding protein 3 (GATA3) and singnal transducer and activator of transcription 6 (STAT6) are the main regulatory factors that guide CD4^+^ T cells to differentiate into Th2 type inflammation. Previous studies have confirmed that Sirtuin 1 (SIRT1) is a predictive target gene for miR‑155‑5p.^20^ SIRT1 is a crucial factor in guiding Th2 type inflammatory response. Thus, after challenging by HDM and RSV activation, the impact of hsa‐miR‐155‐5p on the SIRT1‐mediated pathway in T cells was detected. In our coculture model, the levels of SIRT1, STAT6, and GATA3 in T cells were upregulated following exposure to HDM and RSV, but this upregulation was reversed upon inhibition of hsa‐miR‐155‐5p (Figure 5I,J). Consistent with this, SIRT1 inhibitor also significantly reduced the expression of STAT6 and GATA3 in T cells (Figure 5J). Taken together, these results demonstrated that the exaggerated Th2 inflammatory induced by hsa‐miR‐155‐5p was mediated through SIRT1 pathway in acute asthma exacerbation.

### 2.5 Targeted inhibition of hsa‐miR‐155‐5p blocks the exaggerated Th2 inflammatory in mice with acute asthma exacerbation

**FIGURE 6:**
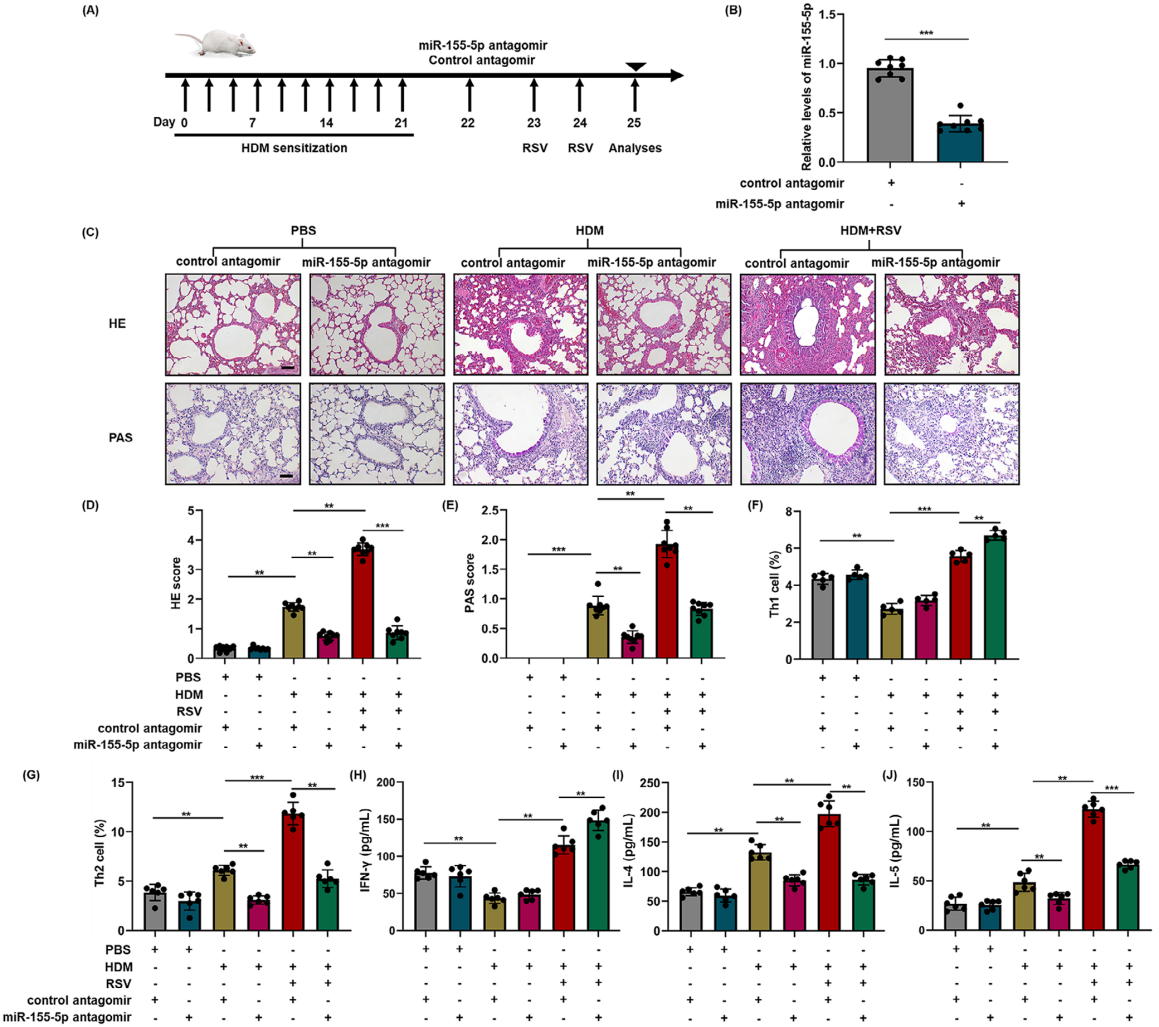
Targeted inhibition of hsa‐miR‐155‐5p blocks the exaggerated pulmonary inflammation in mice with acute asthma exacerbation. (A) The inhibition efficiency of hsa‐miR‐155‐5p antagomir was assessed by qPCR. (B) Schematic for the hsa‐miR‐155‐5p antagomir administration protocol (*n* = 8). (C) H&E staining and PAS staining of lung tissue (*n* = 8), bars = 50 µm. (D and E) Histopathology score. (F and G) The frequency of Th1 cells and Th2 cells in the lungs of mice with acute asthma exacerbation were detected by flow cytometry (*n* = 6). (H–J) The level of IFN‐γ, IL‐4, and IL‐5 in the BALF of mice with acute asthma exacerbation were detected by ELISA after inhibition of hsa‐miR‐155‐5p (*n* = 6). *p < 0.05; **p < 0.01; ***p < 0.001.

To further verify the involvement of hsa‐miR‐155‐5p in mice with acute asthma exacerbation, the expression of hsa‐miR‐155‐5p was effectively suppressed in vivo using an hsa‐miR‐155‐5p inhibitor, as shown in Figure 6A,B. In the mice of acute asthma exacerbation, the degree of inflammatory infiltration in the lungs reduced obviously after blockage of hsa‐miR‐155‐5p. Similarly, the increased secretion of mucus was significantly suppressed after inhibition of hsa‐miR‐155‐5p. However, no obvious changes were observed for the hyperplasia of goblet cells (Figure 6C). In line with the aforementioned findings, the percentage of Th1 cells increased and the enhanced Th2 inflammation decreased significantly after inhibiting hsa‐miR‐155‐5p in mice with acute asthma exacerbation (Figures 6F,G and S5A). Accordingly, the enhanced expressions of IL‐4 and IL‐5 in the lung tissues of acute asthma exacerbation group decreased after hsa‐miR‐155‐5p was inhibited, while the level of IFN‐γ increased (Figure S5B–D). The content of IFN‐γ, IL‐4, and IL‐5 are consistent with the results of mRNA expression (Figure 6H–J). Altogether, these above findings suggest that blockage of hsa‐miR‐155‐5p can significantly suppress the augmented Th2 inflammation in the mice with acute asthma exacerbation.

### 2.6 hsa‐miR‐155‐5p is a potential biomarker for prediction of acute asthma exacerbation

**FIGURE 7:**
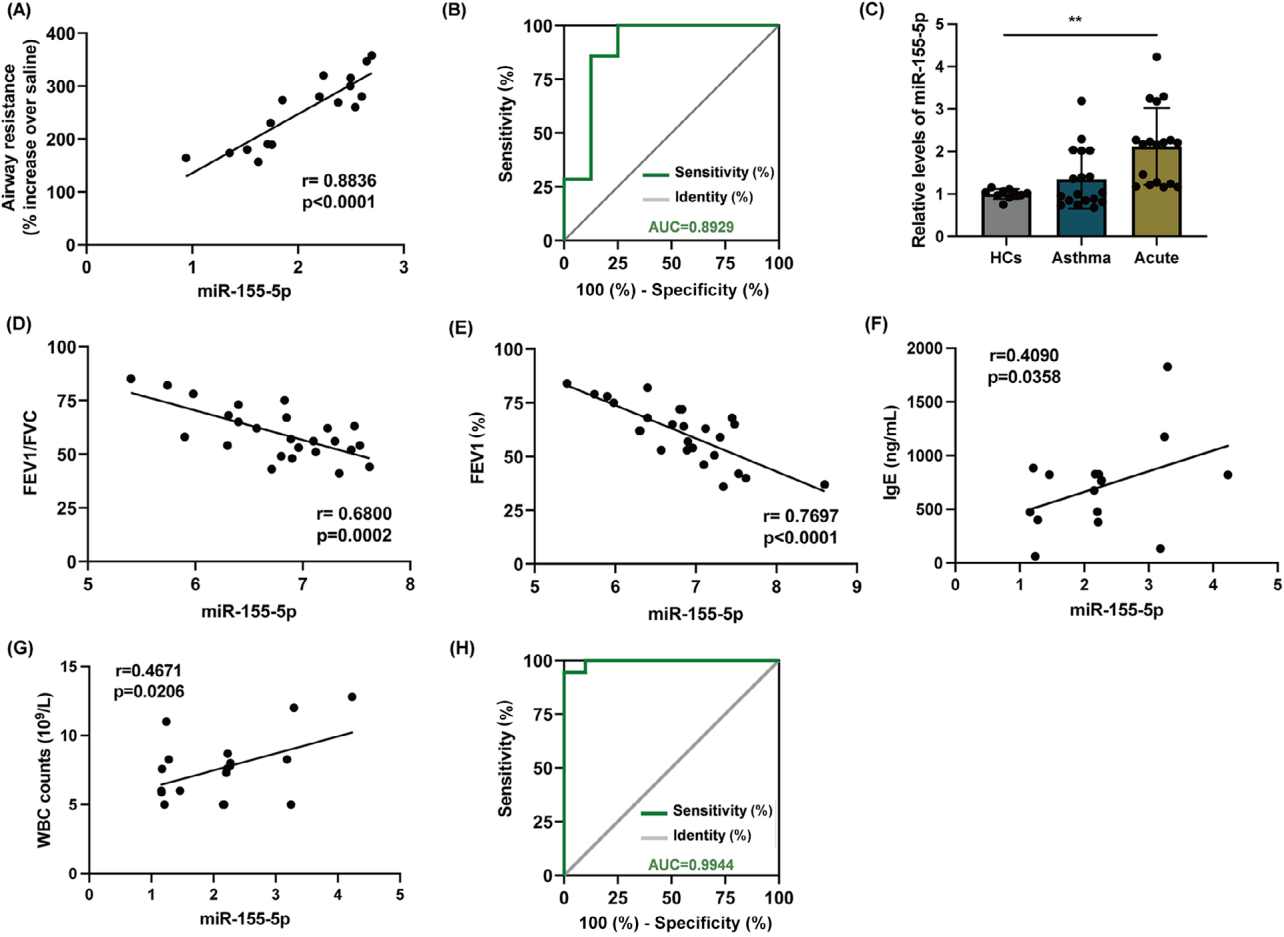
The accuracy of hsa‐miR‐155‐5p in predicting acute asthma exacerbation. (A) The level of hsa‐miR‐155‐5p in the lung tissue of mice with acute asthma exacerbation was positively correlated with AHR. (B) ROC curve of hsa‐miR‐155‐5p between asthma group and acute asthma exacerbation group. (C) The expression of hsa‐miR‐155‐5p in the peripheral blood of asthma patients with acute exacerbation by miRNA RT‐qPCR. (D–G) Correlation analysis of hsa‐miR‐155‐5p with FEV_1_%, FEV_1_/FVC, IgE level, and WBC count, respectively. (F) ROC curve of hsa‐miR‐155‐5p between acute asthma exacerbation and HCs. (F) ROC curve of hsa‐miR‐155‐5p between acute asthma exacerbation and asthma patients. *p < 0.05; **p < 0.01; ***p < 0.001.

Our findings support that hsa‐miR‐155‐5p is highly concentrated in AEC‐Exos and significantly contributes to the enhanced Th2 inflammation observed in mice with acute asthma exacerbation. As a key molecule regulating immune inflammation, hsa‐miR‐155‐5p exerts a vital role in eruptive inflammation. We next determine whether hsa‐miR‐155‐5p could act as a potential biomarker for acute asthma exacerbation. AHR is widely recognized as an effective indicator reflecting the severity of asthma.^18^ Correlation analysis showed that the level of hsa‐miR‐155‐5p in the lungs of mice with acute asthma exacerbation was significantly positively correlated with AHR (Figure 7A). Meanwhile, the level of hsa‐miR‐155‐5p was also significantly positively correlated with the expression of Th2 inflammatory cytokines (IL‐4 and IL‐5) (Figure S6A,B). Furthermore, ROC analysis between asthma group and acute asthma exacerbation group showed that the area under the curve of hsa‐miR‐155‐5p reached 0.8929, 95% confidence interval was 0.7134 to 1.000, *p *= 0.0109, which suggested that hsa‐miR‐155‐5p had a high accuracy in the prediction of acute asthma exacerbation (Figure 7B). Of note, although ROC analysis showed that hsa‐miR‐155‐5p could distinguish between control group and asthma group, its significance (AUC = 0.667) was much smaller than that of acute asthma exacerbation group (*p *= 0.2888) (Figure S6C). Then, in order to further verify the potential of hsa‐miR‐155‐5p as a biomarker of acute asthma exacerbation, a cohort study was conducted including HCs, asthma patients and asthma patients with acute exacerbation, and the demographic characteristics are shown in Table 1. In asthma patients with acute exacerbation, the expression level of hsa‐miR‐155‐5p in the peripheral blood was significantly higher than that in HCs and asthma group (Figure 7C). Furthermore, linear analysis demonstrated a negative correlation between the level of hsa‐miR‐155‐5p in peripheral blood and both FEV1%and FEV1/FVC. Conversely, a positive correlation was observed with the levels of IgE and white blood cell count, essential clinical indicators for diagnosing asthma (Figure 7D–G). Similarly, hsa‐miR‐155‐5p can distinguish the cohort well (AUC = 0.9944, 95% confidence interval 0.9766 to 1, *p *< 0.0001) (Figure 7H). The level of hsa‐miR‐155‐5p also have predictive value in distinguishing between asthma patients with acute exacerbation and asthma subjects (AUC = 0.8646, 95% confidence interval 0.9314–1, *p* < 0.0001). Correlation analysis has also been conducted in asthma group, and the correlation was smaller than that of asthma patients with acute exacerbation (Figure S6D,E). These valuable correlation data strongly indicate that the level of hsa‐miR‐155‐5p in the peripheral blood may represent as a potential biomarker for the prediction and treatment evaluation of acute asthma exacerbation.

**TABLE 1:** Demographic characteristics of acute asthma exacerbation and HCs.

Parameters	Healthy control	Asthma patients	Acute asthma exacerbation patients
Number of subjects	10	18	18
Age	38.50 ± 3.75	39.7 ± 8.7	37 ± 4.07
Male	6	8	11
Female	4	10	7
FEV_1_% predicted	/	0.75 ± 0.21*	0.63 ± 0.25*
FEV_1_/FVC (%)	/	0.72 ± 0.10*	0.66 ± 0.15*
IgE (ng/mL)	/	820.60 ± 15.15*	787.91 ± 16.73*
Feno ppb)	/	40.90 ± 3.19*	38.60 ± 6.67*
White blood cell count (10^9^/L)	6.42 ± 1.91	7.94 ± 2. 71	8.11 ± 1.82*
Neutrophil cell count (10^9^/L)	3.67 ± 1.31	4.95 ± 1.89*	6.64 ± 2.19*
Eosinophil cell count (10^9^/L)	0.19 ± 0.33	0.42 ± 0.37*	0.48 ± 0.39*

Data are presented as mean ± SD.

* *p* < 0.05, asthma versus healthy control or acute asthma exacerbation versus healthy control.

## 3 DISCUSSION

Acute asthma exacerbation is the emphasis and difficulty in the treatment of asthma, which is characterized by outbreak of Th2 inflammation. Although RSV infection is known to be an important trigger, the internal mechanism of acute asthma exacerbation is far from clear.^21^ AECs are the primary cells targeted and first sites of RSV infection, which play a crucial role in triggering and sustaining the exaggerated pulmonary immune response, particularly the increased Th2 inflammation, during acute asthma exacerbation.^22^ Especially, AECs are also known to be involved in the regulation of proliferation, activation, and differentiation of CD4^+^ T cells through AEC‐Exos.^23^ However, the critical engagement of AECs in the T cell‐related immune response during acute asthma exacerbation still needs further verification.^24^ In this study, we demonstrate that AEC‐Exos was involved in the regulation of Th2‐dominated pulmonary inflammation during acute asthma exacerbation after RSV infection. Specially, hsa‐miR‐155‐5p is transferred from AEC‐Exos to CD4^+^ T cells that further induce the enhanced Th2 inflammation during acute asthma exacerbation. Our findings have revealed the molecular mechanisms of the exaggerated Th2 inflammation in acute asthma exacerbation and highlighted the potential targets for the treatment of acute asthma exacerbation after RSV infection.

Acute asthma exacerbation can be triggered by high levels of allergen exposure or incorrect treatment. However, it has been proved that acute asthma exacerbation is induced mainly by viral infection such as RSV, accompanied by allergens.^25^ RSV infection has been established as a significant stand‐alone risk factor for acute asthma exacerbations in children, contributing predominantly to the rising incidence and hospitalization rates among children with asthma.^6^, ^26^ Therefore, deep research is still required to clarify the connection between RSV infection and asthma.

Accumulating evidence suggests that exosomes present in the BALF of asthma patients play a crucial role in modulating inflammatory responses, chemokine, and cytokine production, finally leading to the aggravation of asthma.^27^ Correspondingly, after blockage of exosomes by GW4869, the pathological phenotype of asthma was alleviated, which is accompanied with decreased level of inflammation‐related cytokines.^28^ This study further demonstrated that blockage of exosomes could alleviate AHR and lung inflammation simultaneously in acute asthma exacerbation after RSV infection. Moreover, the markedly enhanced Th2 inflammation is also inhibited after GW4869 intervention in the lungs of mice with acute asthma exacerbation. Accordingly, exosomes are involved in the inducement of acute asthma exacerbation by regulating pulmonary inflammation, especially the enhancement of Th2 inflammation. Similar to our findings, previous studies have also shown that exosomes derived from mast cells can release soluble mediators to stimulate the production of IL‐6 and IL‐8 from recipient cells, which further aggravates lung inflammation of asthma patients.^29^ Additionally, in the mouse model of asthma, blockage of exosomes can inhibit the infiltration of inflammatory cells, reduce the secretion of Th2‐related cytokines, and alleviate the enhanced AHR.^30^ It is interesting that the level of IFN‐γ remained relatively stable compared with the varying levels of IL‐4, IL‐5, and IL‐13, despite a slight increase in the number of Th1 cells and level of IFN‐γ in the lungs during acute asthma exacerbation. The exaggerated Th2 inflammation is the dominant type of inflammatory response in acute asthma execration, which can even far surpass and counteract Th1 inflammation. Correspondingly, when exosomes were blocked by GW4869, our results showed that GW4869 has a more significant inhibitory effect on Th2 inflammation. Similarly, Zhang et al.^31^ also reported that the levels of serum IgE and inflammatory cytokines, such as IL‐4, IL‐5, and IL‐13, in the lung were decreased after GW4869 treatment. However, further exploration is needed to investigate the specific effects of GW4869 on different types of T cells in acute asthma exacerbation needed.

As the first defense barrier of respiratory system against external pathogens, AECs regulate the immune response of T cells through a series of complex processes, including antigen presentation, cytokine release, and so on.^32^, ^33^ It is particularly intriguing that the activation of T cells is sensitively regulated though AEC‐Exos. It has proven that AECs can induce the activation of CD4^+^ T cells directly in an antigen‐specific way by releasing exosomes.^34^ The AEC‐Exos carrying MHC‐II molecules can specially bind with the surface receptors of CD4^+^ T cells.^35^ In parallel, during the information exchange between AECs and dendritic cells, AEC‐Exos can also present antigens to dendritic cells to activate the functional characteristic of dendritic cells. These preexisting findings indicate that AEC‐Exos may be the main reason for the crosstalk between epithelial cells and immune cells during the development of asthma.^36^ Based on this, our results also demonstrate that, after HDM stress combined with RSV infection, AEC‐Exos induce the proliferation and differentiation of CD4^+^ T cells effectively, which is Th2 cells dominated. The slight increase of IFN‐γ may be partly initiated by the antiviral response in the lungs that still need further verification.

Previous studies have shown the difference of composition in exosomes during various diseases, which makes them more likely to serve as possible diagnostic biomarkers, predictors of disease activity and progression, and potential tools for effective and decisive therapeutic strategies.^37^ To identify the new potential disease biomarkers, capable of defining the severity of the asthma, this study was designed to validate the potential of exosomal microRNAs as hypothetical markers that could be used in early risk diagnosis and optimal treatment targets for asthma. Interestingly, our results showed that, the exosome diameter in the HDM‐treated group is slightly different from that in the control group. Previous studies have shown that the size of exosome is related to lecithin/cholesterol rate and membrane thickness.^38^ The size of exosome could further affect the uptake efficiency of target cells. However, the influence of HDM on the diameter of exosome is still obscure which need to be further investigated.

As an important substance carried by exosomes, miRNAs are delivered from maternal cells to effector cells to regulate the expression of critical target genes. This process ultimately impacts the physiological and pathological responses of effector cells.^39^, ^40^ Notably, the contents of miRNA in exosomes are relatively high compared with other components (protein, mRNA, lipid, and DNA), as exosomes would protect miRNAs from degradation. Consequently, the abundant miRNA endows exosomes multiple regulative effects on recipient cells.^41^ It has been shown that miRNAs in exosomes is a key intercellular messenger in lung that can serve as a regulator of intercellular communication, mediates of immune, untimely promoting the progression of asthma.^42^, ^43^ In this study, we have found that the expression of hsa‐miR‐155‐5p in AEC‐Exos significantly increased after exposure to HDM stress and RSV infection, leading to the activation of T cells and the enhanced differentiation of Th2 cells. Elevated levels of hsa‐miR‐155‐5p were also observed in the lungs of mice experiencing acute asthma exacerbation. Consequently, targeted inhibition of hsa‐miR‐155‐5p attenuated the exaggerated pulmonary inflammation significantly, accompanied by increased expression of IFN‐γ. Parallel studies also found that the deficiency of hsa‐miR‐155‐5p in the classic Th2‐domintated asthma model can effectively block the activation of Th2 cells, reduce the secretion of Th2 cytokines, and decrease the infiltration of pulmonary eosinophils.^44^

It has also found that exosomes secreted by AECs can be internalized by immune cells to further regulate the immune response by transporting miRNA, proteins, and nucleic acids in exosomes, which plays a crucial role in allergic diseases.^39^, ^45^ By reviewing previous studies, we screened for nine miRNAs that could regulate T cells and inflammatory responses in asthma. Our study found that the contents of hsa‐miR‐155‐5p in AEC‐Exos increased significantly after RSV infection, consequently influencing the progression of lower respiratory tract inflammation. Previous studies have also shown that exosomal hsa‐miR‐155‐5p drives widespread macrophage inflammation in acute lung injury through MSK1/p38–MAPK Axis.^46^ It has also found that hsa‐miR‐155‐3p is functionally relevant in the immune context as a proinflammatory regulator in multiple immune cells, including dendritic cells, macrophages, T cells, and astrocytes.^47^ hsa‐miR‐210 is one of the important molecules that involved in the growth and metastasis of tumor cells.^48^ Upregulated expression of hsa‐miR‐210‐5p and hsa‐miR‐210‐3p suppressed AKT signaling pathway and promoted autophagy or epithelial–mesenchymal transition process.^49^ hsa‐miR‐21 is one of the most well‐studied miRNAs and is associated with inhibiting the invasion of various cancer cell types. It has also been reported that miR‐21‐3p could regulate PI3K/Akt and ERK1/2 signaling during the activation process of fibroblasts and endothelial cells.^50^ Likewise, an increase of miR‐21‐5p was observed in MSC‐EV, which can modulate the activity of immune cell activity and progression of cancer cells.^51^ In addition, we have also identified a significant correlation between let‐7a and inflammatory response of various human tumors.^45^ Specifically, let‐7a‐5p was found to regulate the activation and recruitment of T cells and participate in neuroimmune interactions.^52^

Of note, as an important natural carrier of noncoding RNA (lncRNA and miRNA), studies have shown that the expression profiles of miRNA in the serum exosomes can be used as biomarkers in several diseases.^53^, ^54^ Additionally, exosomes can be more easily obtained from various biofluids, making them a noninvasive approach for the detection of human pathologies.^55^ Accordingly, our results further demonstrated that hsa‐miR‐155‐5p is a potential biomarker for the prediction of the acute exacerbation of asthma. The increased expression of hsa‐miR‐155‐5p in the peripheral blood of asthma patients with acute exacerbation is negatively correlated with FEV_1_% and FEV_1_/FVC, respectively. Although these results indicated the potential of hsa‐miR‐155‐5p as a biomarker of acute asthma exacerbation, there are also some limitations in this study. The first one is that the existing relevance evaluations from animal models may be different from clinical data to a certain extent. Besides, the sample size of our cohort study is relatively small, not comprehensive enough, which should be improved in our subsequent study. In parallel, previous studies have also identified that hsa‐miR‐155‐5p in the exosomes is a potential biomarker for the diagnosis, monitoring, and treatment of PC.^56^ Therefore, hsa‐miR‐155‐5p is potential biomarker for the prediction and diagnosis of several diseases, including cancer and respiratory diseases. Our findings indicated that the expression of hsa‐miR‐115‐5p was notably elevated in asthma patients with acute exacerbations compared with both healthy controls (HCs) and stable asthmatic patients, indicating its potential practicality in predicting asthma severity. The miRNA levels in peripheral blood are easier to detect than other tests, providing a better and faster way to predict acute asthma exacerbation.

In recent years, SIRT1 has been recognized as a key a target gene of miR‐155 participating involved in inflammatory response.^57^ SIRT1 plays a critical role in regulating both metabolism and immune response. Studies on SIRT1 have mainly focused on the cell cycle, aging, and metabolism.^20^, ^58^ It has been reported that the activation and differentiation of T cells was regulated by SIRT1 through interactions with several target substrates. Consequently, activation of SIRT1 can directly upregulate the expression of phosphorylated STAT6 and GATA3, which are Th2 cell specific transcription factor.^59^ Similarly, Liu and coworkers^60^ also reported that SIRT1 induces the differentiation of CD4^+^ T cells by mediating the production of cytokines, bridging innate immune signals to adaptive immune responses. Our results showed that, during acute asthma exacerbation, hsa‐miR‐155‐5p in AEC‐Exos regulates the differentiation of CD4^+^ T cells into Th2 type inflammation through the SIRT1 pathway, which was mediated through enhanced expression of STAT6 and GATA3. However, the specific internal mechanism of hsa‐miR‐155‐5p and SIRT1 pathway during acute asthma exacerbation still needs in‐depth study. Besides, further analysis is also needed on how the hsa‐miR‐155‐5p–SIRT1 axis affects the function of Th2 cells.

## 4 CONCLUSIONS

To sum up, our data provide an insight that AEC‐Exos was involved in the exaggerated pulmonary inflammation of acute asthma exacerbation. Concretely, hsa‐miR‐155‐5p was transported from AEC‐Exos to CD4^+^ T cells through SIRT1‐mediated pathway, which further induce the enhanced Th2 inflammation in acute asthma exacerbation after RSV infection. Understanding the role of hsa‐miR‐155‐5p in AEC‐Exos during acute asthma exacerbation would provide new directions and approaches for the early diagnosis and treatment of acute asthma exacerbation.

## 5 MATERIALS AND METHODS

### 5.1 Mice

All animal studies were approved by the Animal Care and Use Committee of Central South University (No. 2020sydw0305). SPF BALB/c mice (6–8 weeks old, male) were kept in controlled barrier conditions in air‐filtered units with regulated temperature, following a 12‐h light–dark cycle and provided with unlimited access to food and water.

### 5.2 Sensitization and challenge protocols

The HDM‐induced asthma model was created based on our earlier work as described in reference.^61^ Acute asthma exacerbation was induced after RSV‐A2 infection (1 × 10^6^ pfu) by intranasal instillation at 48 and 72 h after the last HDM stress.^62^ GW4869 (2.5 mg/kg; Sigma, USA) was used to block exosomes through tail vein infection 24 h after the last HDM stress.^63^ To inhibit hsa‐miR‐155‐5p, hsa‐miR‐155‐5p antagomir (20 nmol in 40 µL saline; RiboBio) was intranasally instilled 24 h after the last HDM stress,^64^ while the same dose of control antagomir was given for the control group.

### 5.3 Collection of peripheral blood samples

The protocol received approval from the Ethics Review Committee of Xiangya Hospital No.2020KT‐52, and all the subjects provided written informed consent. A total of 46 participants were selected from the Respiratory Department and the Medical Examination Center of Xiangya Hospital, Changsha, including asthma patients (*n* = 18), asthma patients with acute exacerbation (*n* = 18), and HCs without asthma or other acute or chronic diseases (*n* = 10). The criteria for including asthmatic patients consist of the following conditions, as outlined in more detail elsewhere^65^: adults aged 25–70 years; meet the diagnosis of asthma according to GINA (symptoms such as wheezing, shortness of breath, chest tightness, and cough, along with a positive response to either bronchodilator reversibility test or bronchostimulation test); it is not accompanied by other chronic diseases (such as diabetes, cardiovascular disease) or other acute or chronic respiratory diseases (Figure S7). The demographic characteristics of patients with asthma and healthy volunteers are presented in Table 1. Specially, acute asthma exacerbation was defined as the gradual increase of shortness of breath, cough, or wheezing symptoms in asthma patients after exposure to external allergens, and the need to change the treatment plan.^66^ The control group was in the same age and gender without asthma definition or other acute or chronic diseases, including smoking controls and nonsmoking controls.

All participants underwent a survey using a questionnaire that included general information, asthma symptoms, other respiratory diseases, and the outcomes of pulmonary function tests. The questionnaire investigated all subjects, covering general conditions, clinical symptoms of asthma, other respiratory diseases, and results from pulmonary function tests. For our analysis, lung function phenotypes were used included the spirometric values of FEV_1_ and the ratio of FEV_1_ to the FVC. Moreover, peripheral blood samples were collected from all recruiters. Strict quality control measures were implemented. Certified staff conducted all interviews and examinations. Moreover, regular feedback about the quality of their performance was given to each field worker during data collection, and retraining was undertaken when necessary.

### 5.4 Flow cytometry

Lung single cell suspension was prepared as outlined in the previous study for flow cytometry analysis.^67^ At first, live/dead (L/D) aqua (423101; BioLegend, San Diego, CA) viability dye was used to exclude dead cells. Next, the cells were labeled on the surface with a combination of the following antibodies: anti‐CD45 (553079; BD Bioscience), anti‐CD11B (101216; BioLegend), anti‐CD11C (550261; BD Biosciences), anti‐LY‐6G (127613; BioLegend), anti‐Siglec‐F (155527; BioLegend), anti‐CD4 (100411; BioLegend), and isotype controls were incubated to detect different cell subpopulations. Following this, cells were fixed and permeabilized with the 1× Fixation/Permeabilization buffer (BD Biosciences). To conduct intracellular cytokine staining, cells were treated with 50 ng/mL phorbol 12‐myristate 13‐acetate and 1 mg/mL ionomycin for 5 h, with the addition of 3 mg/mL brefeldin A during the final 2 h of in vitro stimulation. Ultimately, the cells were internally stained with anti‐IFN‐γ (163503; BioLegend) and anti‐IL‐4 (504119; BioLegend). Data collection was conducted using FACS Canto flow cytometry and then analyzed using FlowJo software.^68^, ^69^ The gating strategies were conducted based on previous studies with slight alterations.^70^, ^71^ CD45^+^CD4^+^IFN‐γ^+^ is the gating strategy used to define Th1 cells. CD45^+^CD4^+^IL‐4^+^ is defined as the gating strategies of Th2 cells. A CD11B versus Ly‐6G plot gated on CD45^+^cells was utilized to identify neutrophils that are positive for both CD11B and Ly‐6G markers. A plot of CD11C versus Siglec‐F gated on CD45^+^ cells was used to discriminate from eosinophils (CD11C^−^Siglec‐F^+^).

### 5.5 Cell culture and treatment

AECs were acquired from Lifeline Cell Technology (Frederick, MD, USA) and cultured following the procedures outlined in reference.^72^ To construct HDM‐stressed AECs, AECs were stimulated with HDM (75 µg/mL) for 24 h. Subsequently, some AECs were infected with RSV‐A2 for 2 h. To block hsa‐miR‐155‐5p, hsa‐miR‐155‐5p inhibitor (25 nM; RiboBio) was pretreated with AECs for 24 h.

### 5.6 RNA extraction, RT‐PCR, and quantitative RT‐PCR

Total RNA from mouse lung tissues or AECs was in the lung tissues of mice or AECs was extracted by TRIzol reagent (Invitrogen) and quantified by SmartSpecTM Plus spectrophotometer (Bio‐Rad, USA). PCR and qPCR were performed following the protocol of the PrimeScript RT Master Mix Kit (Takara, Japan).^73^ The mRNA expression was normalized by comparing the copy numbers of β‐actin, and the results were expressed as a fold‐change relative to control samples. The primers sequences are provided in Table S1.

### 5.7 Extraction and identification of exosomes

The exosomes derived from AECs were separated by differential ultracentrifugation as previously mentioned.^74^ Briefly, the conditioned medium of AECs was centrifuged for 15 min (4°C, 3000×*g*) to remove cell fragments. Then, the centrifuge products was centrifuged for another 30 min (4°C, 2000×*g*) and 45 min (4°C, 10,000×*g*) to further remove the large vesicles, and filtrated through 0.45 µm membrane. After that, the pellet was washed with 10 mL of precooled PBS heavy suspension and centrifuged for 70 min (4°C, 100,000×*g*). The exosomes were examined for their size distribution and nanoparticle concentration using TEM and Flow NanoAnalyzer (FNA). As per the guidelines, the BCA protein detection kit (Thermo, USA) was utilized for determining the protein concentration of the exosomes. The expressions of CD63 (EXOAB‐CD63A‐1; SBI, USA), CD9 (ab236630; Abcam, USA) CK19 (ab76539; Abcam), and β‐actin (ab8226; Abcam) were separated by western blot. Briefly, protein samples were separated by SDS‐PAGE and transferred onto a nitrocellulose filter membrane. The membranes were subsequently incubated overnight with the primary antibody in a 2.5%milk/TBST buffer. Following this, the PVDF membranes were then incubated with horseradish peroxidase (HRP)‐conjugated anti‐mouse or anti‐rabbit IgG (Abcam) for 1 h.

### 5.8 Exosomes labeling and cellular uptake

A total of 5 µg of AECs‐exosomes were mixed with 1 mL of Diluent C (Sigma) and subsequently stained with 6 µL of PKH67 (green fluorescence; Sigma). After incubation at 37°C for 2 h, 200 µL PKH67‐labeled AEC‐Exos was added to recipient cells for another 24 h. In the end, the confocal microscope was utilized to observe PKH67‐labeled exosomes within recipient cells.

### 5.9 Statistical analysis

This study requires a minimum of three repetitions for each experiment. GraphPad Prism 9.0 software is used to plot the experimental data, and t‐test is used to analyze and compare the data of two independent samples. One‐way ANOVA is used for multigroup comparison. Statistical significance was determined for *p* values less than 0.05(*), 0.01(**), or 0.001(***).

## AUTHOR CONTRIBUTIONS

Ye Yao and Yu Yang carried out the experiments, analyzed and interpreted the data, and drafted the manuscript. Ye Yao, Yu Yang, Ming Ji, Lin Yuan, and Xizi Du collected clinical samples. Leyuan Wang, Xinyu Wu, Kai Zhou, and Weijie Wang performed the experiments and statistical analysis. Yang Xiang, Xiangping Qu, Huijun Liu, Xiaoqun Qin, and Chi Liu analyzed and interpreted the data, provided the project funding, and revised the manuscript. Chi Liu analyzed and interpreted the data, revised the manuscript, and finally approved the version of the manuscript for publication. All authors provided critical feedback and helped shape the research, analysis, and manuscript. All authors have read and approved the final manuscript.

## CONFLICT OF INTEREST STATEMENT

The authors declare no conflict of interest.

## ETHICS STATEMENT

The whole study design and protocols were approved by the Ethics Review Committee of Xiangya Hospital, China (approval number: 2020KT‐52) and the Animal Care and Use Committee of Central South University (approval number: No.2020sydw0305). Written informed consent was obtained from all participants.

## Supporting information

Supporting Information

## Data Availability

All data relevant to the study are included in the article or uploaded as Supporting Information. All other relevant data are available from the lead contact upon reasonable request.
