# Supplementary material for: Airway epithelial‐derived exosomes induce acute asthma exacerbation after respiratory syncytial virus infection
Source: MedComm (2020). 2024 Jun 27;5(7):e621. doi: 10.1002/mco2.621 (PMC11208743; doi:10.1002/mco2.621)
Supplement: Supplementary file 1 — Supporting Information [file MCO2-5-e621-s001.docx]

**Airway epithelial-derived exosomes induce acute asthma exacerbation after respiratory syncytial virus infection**

Ye Yao^1,2#^, Yu Yang^1,2#^, Ming Ji^2^, Qingwu Qin^3^, Kun Xu^4^, Zhenkun Xia^5^, Huijun Liu^2^, Lin Yuan^2^, Yunchang Yuan^5^, Ling Qin^2,6^, Xizi Du^2^, Leyuan Wang^2^, Kai Zhou^2^, Xinyu Wu^2^, Weijie Wang^2^, Bei Qing^5^, Yang Xiang^2^, Xiangping Qu^2^, Ming Yang^7^, Xiaoqun Qin^2^, Chi Liu^1,2,6*^

**Supplemental materials**

**ELISA assay**

The levels of IFN-γ, IL-4 and IL-5 in the supernatants of CD4^+^ T cells were measured by ELISA according to the manufacturer's protocol (BioLegend, USA).

**Total RNA purification from AEC-Exos**

Total exosomal RNA was purified with the ‘Exosome RNA Purification Kit’ (SBI, USA) following the manufacturer's instructions.

**miRNA RT-qPCR**

Preparation and quantification of miRNA were carried out according to previous publication [26].

**Measurement of lung function in mice**

As previously described [24], airway resistance was measured using a direct plethysmography (Biosystems XA, Buxco Electronics, USA).

**Histopathology of lung tissue**

Parafﬁn-embedded lung sections were stained with hematoxylin and eosin (HE), Glycogen Periodic Acid Schiff (PAS) and Masson, respectively. Histopathological changes (airways inﬂammation, mucus secretion and collagen deposition) were scored blindly according to morphological criteria, with reference to previous publication [24].

**Bronchoalveolar lavage fluid collection and cell counting**

Bronchoalveolar lavage fluid (BALF) was collected as described before. In brief, BALF was centrifuged to collect cellular infiltrate. Cells in BALF were then plated on a glass side and quantified using a hemocytometer. Differential leukocyte counts were determined based on morphological criteria by light microscopy (x100) after Giemsa Staining [29].

**T cells proliferation Assay**

EdU proliferation Assay and Cell Counting Kit-8 (CCK-8) detection were used to detect the proliferation of CD4^+^ T cells, respectively. For EdU proliferation detection, the proliferation of CD4^+^ T cells was assessed using the BeyoClick™ 5-ethynyl-2′-deoxyuridine (EdU) Cell Proliferation Kit with TMB (Beyotime, Shanghai, China). Enzyme reader was used to detect the absorbance at 450nm. For CCK-8 detection, CD4^+^ T cells was inoculated into 96 well plates (2000 cells/well) and exposed to 20µl of CCK-8 reagent (Beyotime, Shanghai, China) for continuing 3h. The absorbance at 450nm was examined using a microplate reader.

**T cells derivation Assay**

CD4^+^ T cells from peripheral venous blood was obtained from healthy subjects following the approval for the use of human tissues which was granted by No. 2020KT-52 of the Ethical Committee of Central South University after obtaining written informed consent from individual donors [30]. After isolation, CD4^+^ T cells was stimulated with phorbol 12-myristic acid 13-acetate (50ng/mL; Calbiochem, San Diego, CA) and ionomycin (1g/mL; Calbiochem) at 37℃ for 5 hours in the presence of Brefeldin A (3 mg/mL; BD Bioscience, USA). The derivation of CD4^+^ T cells was detected by cytokines staining which was measured by flow cytometry. Then, cells was fixed and treated with 1x Fixation/Permeabilization buffer (BD Bioscience) and stained with corresponding fluorescent labeled antibodies of IFN-γ (568687, BD Biosciences) and IL-4 (500808, BD Biosciences) [31].

**Western blot analysis**

Western blot was performed as described previously [10]. Briefly, 50 ug protein from HBE cells or CD4^+^ T cells from mice was prepared and separated by 10% or 12% SDS/PAGE. Then, the separated protein was transferred to a polyvinylidene difluoride (PVDF) membrane. Next, the PVDF membrane was incubated with antibody overnight and then incubated with horseradish peroxidase-conjugated secondary antibody. The following antibodies were used to determine the expression of corresponding protein: SIRT1 (sc-74465, Santa Cruz), STAT6 (sc-374521, Santa Cruz), GATA3 (sc-268, Santa Cruz). β-actin (ab8226, Abcam) was used as corresponding controls.

**
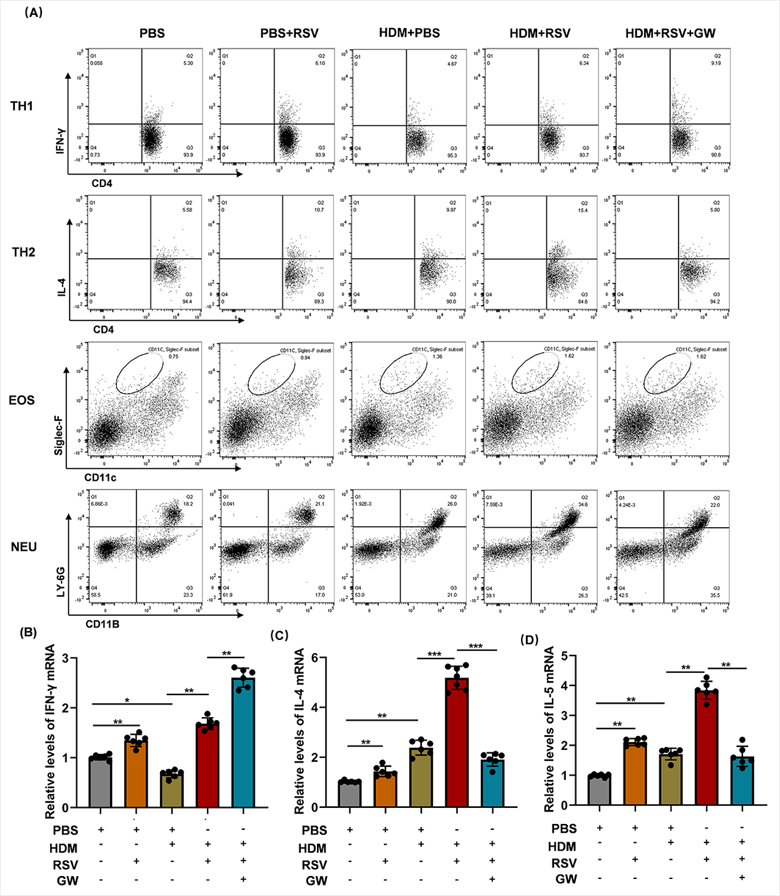
**

**Figure S1.** Involvement of exosomes in the deteriorative pulmonary inflammation in asthmatic mice with acute exacerbation. (A) Scatter plot of Th1, Th2, eosinophils and neutrophils. (B-E) The mRNA expression of IFN-γ, IL-4, IL-5 and IL-13 in the lung tissue were detected by qPCR (n=6). **P*＜0.05; ** *P*＜0.01; ****P*＜0.001.


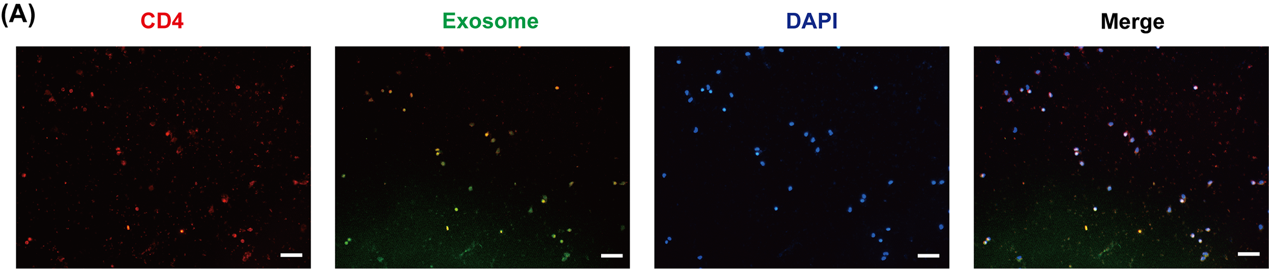


**Figure S2.** Immunofluorescent staining for CD4 and exosome in CD4^+^ T cells.


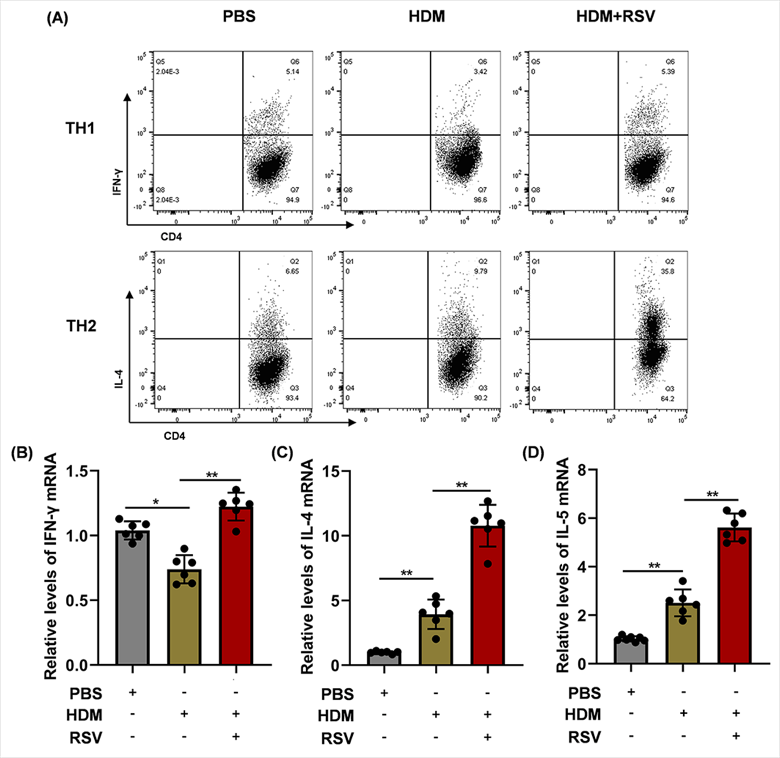


**Figure S3.** The proliferation and differentiation of CD4^+^ T lymphocytes was regulated by AEC-Exos. (A) Scatter plot of Th1 cells and Th2 cells. (B-D) The mRNA expression of IFN-γ, IL-4 and IL-5 was detected by qPCR after co-culture (n=6). **P*＜0.05; ** *P*＜0.01; ****P*＜0.001.


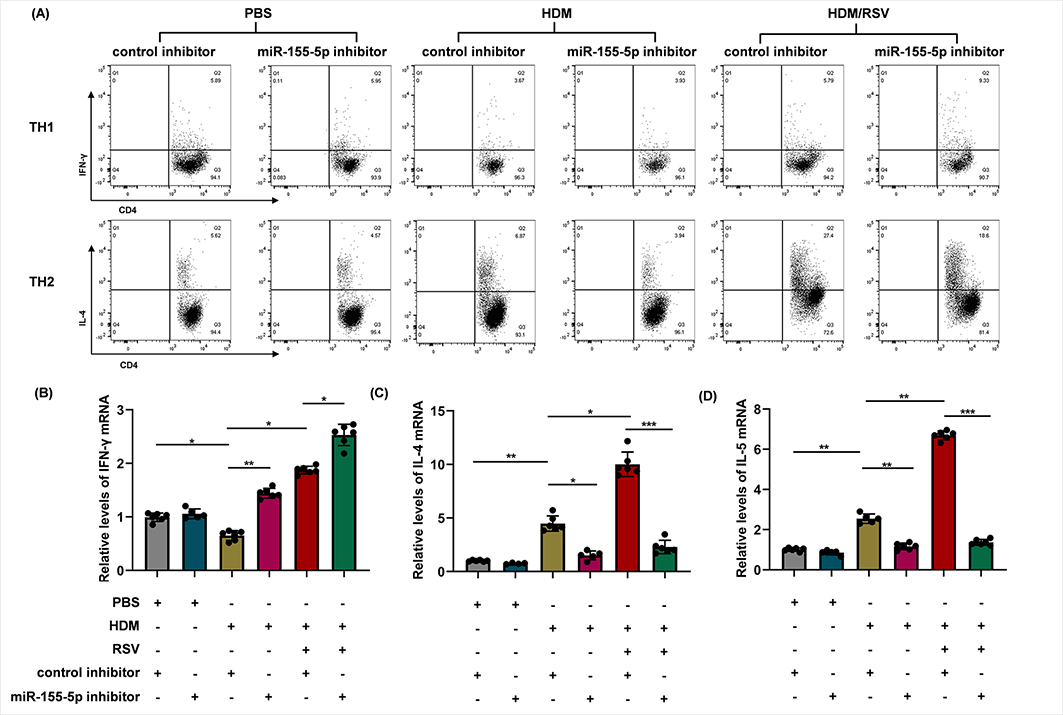


**Figure S4.** The proliferation and activation of CD4^+^ T cells were induced by hsa-miR-155-5p in AEC-Exos**.** (A) Scatter plot of Th1 cells and Th2 cells. (B-D) The mRNA expression of IFN-γ, IL-4 and IL-5 were detected by qPCR after co-culture (n=6). **P*＜0.05; ** *P*＜0.01; ****P*＜0.001.


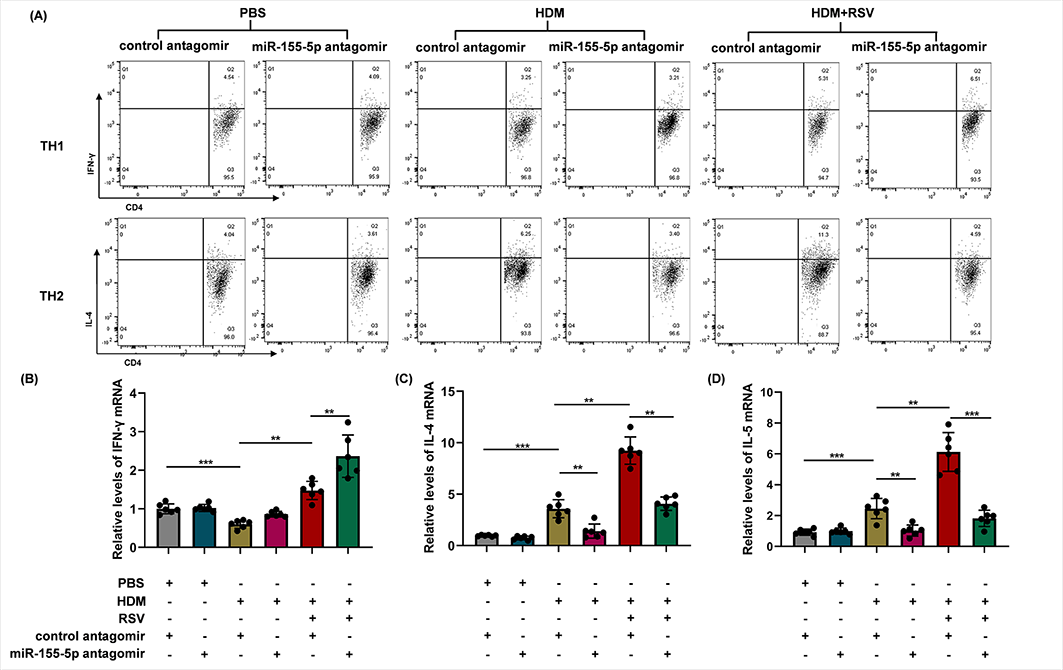


**Figure S5.** Targeted inhibition of hsa-miR-155-5p blocks the exaggerated pulmonary inflammation in asthmatic mice with acute exacerbation. (A) Scatter plot of Th1 cells and Th2 cells after hsa-miR-155-5p antagomir intervention. (B-D) The mRNA expression of IFN-γ, IL-4 and IL-5 after hsa-miR-155-5p inhibition were detected by qPCR (n=6). **P*＜0.05; ** *P*＜0.01; ****P*＜0.001.


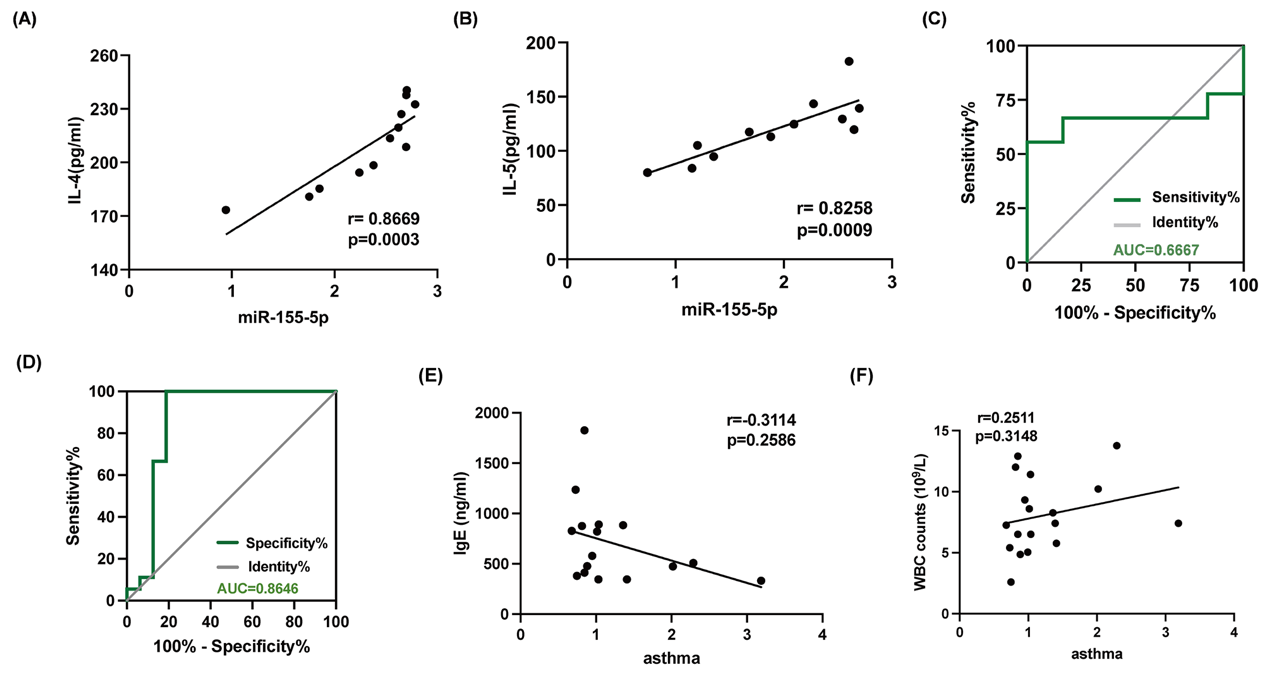


**Figure S6.** The accuracy of hsa-miR-155-5p in predicting acute asthma exacerbation. (A) The level of hsa-miR-155-5p was positively correlated with IL-4. (B) The level of hsa-miR-155-5p was positively correlated with IL-5. (C) ROC curve of hsa-miR-155-5p between control group and asthma group. (D) ROC curve of hsa-miR-155-5p between asthma acute exacerbation group and asthma group. (E-F) Correlation analysis of hsa-miR-155-5p with FEV1%, FEV1/FVC, IgE level and WBC count in asthma group, respectively.


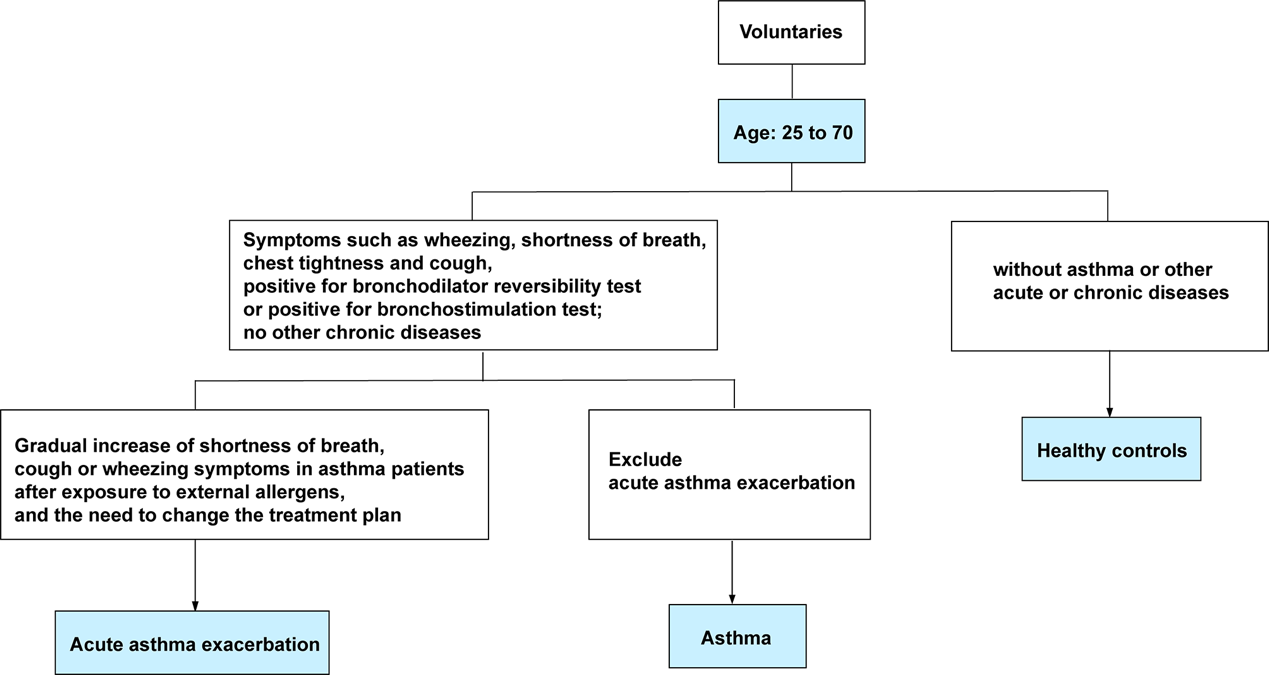


**Figure S7**. Flow chart of the inclusion criteria for asthma patients and health controls.

**Table S1.** Primer sequence of genes for PCR and qPCR.

| Primer genus | Gene |  | Primer sequence |
| --- | --- | --- | --- |
| Mouse | β-actin | Forward: TTGCAGCTCCTTCGTTGCC | |
|  |  | Reverse: GACCCATTCCCACCATCACA | |
| Mouse | IL-4 | Forward: TACCAGGAGCCATATCCACGGATG | |
|  |  | Reverse: TGTGGTGTTCTTCGTTGCTGTGAG | |
| Mouse | IL-5 | Forward: AGAGAAGTGTGGCGAGGAGAGAC | |
|  |  | Reverse: CCATTGCCCACTCTGTACTCATCAC | |
| Mouse | IFN-γ | Forward: CTGGAGGAACTGGCAAAAGGATGG | |
|  |  | Reverse: GACGCTTATGTTGTTGCTGATGGC | |
| Human | β-actin | Forward: TTGCAGCTCCTTCGTTGCC | |
|  |  | Reverse: GACCCATTCCCACCATCACA | |
| Human | IL-4 | Forward: GCAGAATCCGCTCAGCATCCTC | |
|  |  | Reverse: CGTACTCTGGTTGGCTTCCTTCAC | |
| Human | IL-5 | Forward: CTTGGAGCTGCCTACGTGTATGC | |
|  |  | Reverse: GAACAGGAATCCTCAGAGTCTCATTGG | |
| Human | IFN-γ | Forward: CTGACTTGAATGTCCAACGCAAAGC | |
| Human  Human  Human | SIRT1  STAT6  GATA3 | Reverse: CGACCTCGAAACAGCATCTGACTC  Forward: TAGCCTTGTCAGATAAGGAAGGA  Reverse: ACAGCTTCACAGTCAACTTTGT  Forward: GCCCCTCATTAAGCCCAAG  Reverse: TTGTGGTGGTCTGACAGTTCG  Forward: GTTCCGCCACTTGCCAATG  Reverse: TGGATCTCCCCTACTCGGTG | |
